# ‘AAC Isn't a Take It or Leave It’: The Augmentative and Alternative Communication Training Experiences of Australian Speech‐Language Pathologists Working in Paediatrics

**DOI:** 10.1111/1460-6984.70111

**Published:** 2025-08-23

**Authors:** Clancy Conlon, Robyn Preston, Barbra Zupan

**Affiliations:** ^1^ Central Queensland University, School of Health, Medical and Applied Sciences College of Health Sciences, Speech Pathology Rockhampton Australia; ^2^ Central Queensland University, School of Health, Medical and Applied Sciences College of Science and Sustainability, Public Health Rockhampton Australia

**Keywords:** augmentative and alternative communication, speech‐language pathologists, training

## Abstract

**Background:**

Augmentative and alternative communication (AAC) is a core area of practice for Australian speech‐language pathologists (SLPs); however, there is no current literature describing the state of AAC training in Australia.

**Aim:**

Therefore, this study aimed to investigate the training experiences and needs of Australian SLPs in AAC.

**Method and Procedures:**

This study followed a sequential‐explanatory mixed methods approach. First, 205 SLPs completed a quantitative online survey. Next, 16 SLPs participated in a one‐on‐one, semi‐structured interview, which was conducted over Zoom. Results were than triangulated for analysis.

**Outcomes and Results:**

Overall, SLPs rated their university training in AAC as poor, and this did not differ based on time spent in the workforce. SLPs felt that current students should be receiving comprehensive training in AAC at university, inclusive of theory and practice. SLPs reported accessing a range of post‐professional training, but the most common training formats did not align with their training preferences, which included practical, face‐to‐face training with a presenter who is knowledgeable and passionate about AAC.

**Conclusions and Implications:**

Given that AAC has been recognised as a practice area in Australia since 2012 and AAC users are present on most paediatric SLP caseloads, better quality training needs to be made accessible throughout Australia. This is particularly pertinent for university programmes that are responsible for training the future SLP workforce.

**WHAT THIS PAPER ADDS:**

*What is already known on this subject*

- Speech‐language pathologists (SLPs) internationally report limited training in AAC while at university. Minimal research has been conducted on the training of SLPs in AAC in Australia.

*What this study adds to existing knowledge*

- Australian SLPs report inadequate AAC training at university and feel that SLP students require explicit teaching in this area in addition to practical experiences. Qualified Australian SLPs want more training in all content areas relating to AAC.

*What are the potential or actual clinical implications of this work?*

- University stakeholders should consider how they are currently preparing SLPs to enter the contemporary workforce, which has a high proportion of AAC users. Employers of SLPs should provide a range of training options, including ongoing supervision/mentoring.

## 1 Introduction

Speech‐language pathology is a quickly growing profession in Australia, with 6044 registered, practising members of Speech Pathology Australia in 2015 compared with 12 185 in 2023 (Speech Pathology Australia 2023). The majority of these speech‐language pathologists (SLPs) are employed in private practice, followed by public health and other public sectors such as education, not‐for‐profit organisations and universities (Speech Pathology Australia 2023). To become a SLP in Australia, students must complete a 4‐year bachelor's or 2‐year master's programme that is accredited by Speech Pathology Australia (Speech Pathology Australia 2024b). As of 2023, there were 45 bachelor's or master's level SLP programmes in Australia, offered across 25 universities. Each university programme is individually accredited by Speech Pathology Australia on an ongoing basis against the Accreditation Standards (Speech Pathology Australia 2024a), which align with the Professional Standards for Speech Pathologists in Australia (Speech Pathology Australia 2020b). Once qualified, to maintain registration with Speech Pathology Australia, SLPs must complete a minimum of 20 hours of professional learning each year including a minimum of 2 hours of cultural learning and 2 hours of professional support (Speech Pathology Australia 2024c).

While SLPs in Australia work in a range of practice areas, augmentative and alternative communication (AAC) as a practice area for SLPs in Australia has a long and complex history which has shaped the way Australian SLPs view, approach and engage with AAC in workplaces today. The state of SLPs’ knowledge and skills in AAC was initially investigated in 1998 when Balandin and Iacono published the results of a national survey representing 971 Australian SLPs. They identified that the majority (84%) of SLPs either occasionally/sometimes or frequently recommended AAC despite an alarming number also reporting limited to no knowledge of unaided (37%), low‐technology (42%) and high‐technology (50%) systems. At this time, the Competency‐Based Occupational Standards (CBOS) for SLPs in Australia were under review, with the first edition released in 2001 (Speech Pathology Australia 2001). This iteration of CBOS acknowledged AAC within the definition of communication but indicated that SLPs may require additional training or should seek support from other SLPs working in AAC as a ‘relevant field’. AAC was not formally recognised as an individual practice area in Australia until the introduction of multimodal communication (MMC) in the CBOS by Speech Pathology Australia in 2011 (Speech Pathology Australia 2011). Following the introduction of MMC as a practice area, Speech Pathology Australia (2012) released the Augmentative and Alternative Communication Clinical Guideline outlining the need for theoretical knowledge and clinical experience to be incorporated into speech pathology courses. These guidelines could be viewed as a monumental turning point for AAC in Australia, as university programmes across the nation were then required to graduate SLPs with knowledge and skills in AAC.

In 2020, CBOS was replaced with the Professional Standards, which detail the knowledge, skills and attributes speech pathology students must demonstrate upon graduation and maintain throughout their career (as relevant to their work context) (Speech Pathology Australia 2020b). Once again, this document stipulated that ‘speech pathologists work towards optimising communication, for a range of purposes and across different contexts including … augmentative and alternative communication’ (Speech Pathology Australia 2020b, 6). Based on this history, it could be assumed that any SLP who has graduated from an Australian university in the last 10 years should have received adequate training in AAC. However, there has been limited research completed on the training of Australian SLPs in AAC since the national survey conducted in 1998 (Balandin and Iacono 1998). In 2009, Australian SLPs working in early childhood described mixed skill levels when interviewed and felt they needed more time to update their knowledge about AAC (Iacono and Cameron 2009). In 2019, Australian SLPs working with AAC users reported accessing a range of post‐professional AAC training, including internal training, informal mentoring, Pragmatic Organisational Dynamic Display (PODD) introduction, Picture Exchange Communication System (PECS) and Key Word Sign presenter training (Moorcroft et al. 2019); however, university training experiences were not reported. A specific investigation of the training of SLPs in Key Word Sign (Wen and Sutherland 2022) identified that only 9.67% of respondents received training at university, and the remainder relied on post‐professional training. This literature does not provide a comprehensive picture of the state of university or post‐professional training in AAC for Australian SLPs.

The importance of understanding the state of AAC training has been recognised through a recent burst of literature on the topic. Although the United States is the main source of this research, studies published internationally have highlighted the unique and complex nature of this practice area in relation to training needs (Chua and Gorgon 2018; Dada et al. 2017; Pampoulou et al. 2018; Sauerwein and Burris 2022). Unlike practice areas such as speech, language, or voice, where clients may have difficulties in just that single area (e.g., a child with a phonology delay or a teacher with vocal nodules), AAC users always present with needs across multiple areas. In a survey of New Zealand SLPs servicing clients who could not rely on speech alone to be heard and understood, paediatric and adult AAC users presented with a range of aetiologies, including intellectual disability, autism spectrum disorder, cerebral palsy, cerebrovascular accidents, amyotrophic lateral sclerosis and traumatic brain injury (Sutherland et al. 2005). In addition to the theoretical and clinical aspects of AAC, working with people with disabilities requires training in a range of disability‐specific practices, such as using appropriate terminology (CommunicationFIRST 2023), providing neuro‐affirming therapy services (DeThorne and Searsmith 2021) and working within a multidisciplinary team (Hunt et al. 2002). Finally, AAC users present with diverse cultural and linguistic backgrounds, meaning that all SLPs working with AAC users also need to understand how to modify their assessment and management practices to support bilingual and multilingual AAC users (Dada et al. 2017). Combining this vast range of knowledge and skills to service a single client requires extensive training and experience.

Prior to the introduction of the National Disability Insurance Scheme (NDIS) in 2013 (Parliament of Australia 2018), people with disabilities in Australia primarily accessed therapy supports through government‐funded and regulated organisations that specialised in providing disability supports. Under this pre‐NDIS model, funding was state‐based, and therefore access to funding and disability services differed for individuals based on their age, disability diagnosis and geographical location. Comparatively, the NDIS is a national scheme for people with disabilities under 65 years of age which supports over 502 413 participants (of which 77 287 are under 7 years) and was estimated to spend 29.2 billion dollars on disability‐related supports in 2022 (National Disability Insurance Scheme 2022). The NDIS promotes choice and control for people with disabilities in relation to the services that they access, and therefore disability services are now provided by the private and not‐for‐profit sectors, of which a large proportion are not NDIS registered (Australian Government 2023). Subsequently, families can access these services utilising NDIS funding, but these organisations are not NDIS regulated. Consequently, any SLP in Australia working in a private practice or non‐government organisation can support any client, in any practice area, including AAC users, regardless of that SLP's experience or training. While the intent of the NDIS is to allow participants to exercise choice and control over their therapy, families and support personnel around Australia report countless barriers to accessing therapy services (Gavidia‐Payne 2020), including finding service providers who have the appropriate expertise and skills (Dickinson and Yates 2023). Given this report and the limited research in this area, this study aimed to investigate the training experiences and needs of paediatric Australian SLPs in AAC. Paediatric SLPs were selected because paediatric AAC users typically form a relatively consistent diagnostic profile with developmental disabilities (Sutherland et al. 2005). In contrast, adult AAC users may have developmental disabilities and/or acquired disabilities necessitating different training in assessment and treatment approaches (Beukelman and Light 2020). Consequently, in line with previous AAC research (Biggs et al. 2022; Iacono and Cameron 2009; Sanders et al. 2021; Simpson et al. 1998), the decision was made to concentrate on SLPs with a paediatric caseload through four research questions:

1. What are the university training experiences of Australian SLPs in AAC?
2. What AAC training experiences should SLP students receive at Australian universities?
3. What are the post‐professional training experiences of Australian SLPs in AAC?
4. What are the training needs of Australian SLPs in AAC?

## 2 Method

This study was approved by the Central Queensland University Human Research Ethics Committee (reference number 23676).

### 2.1 Research Design

**FIGURE 1:**
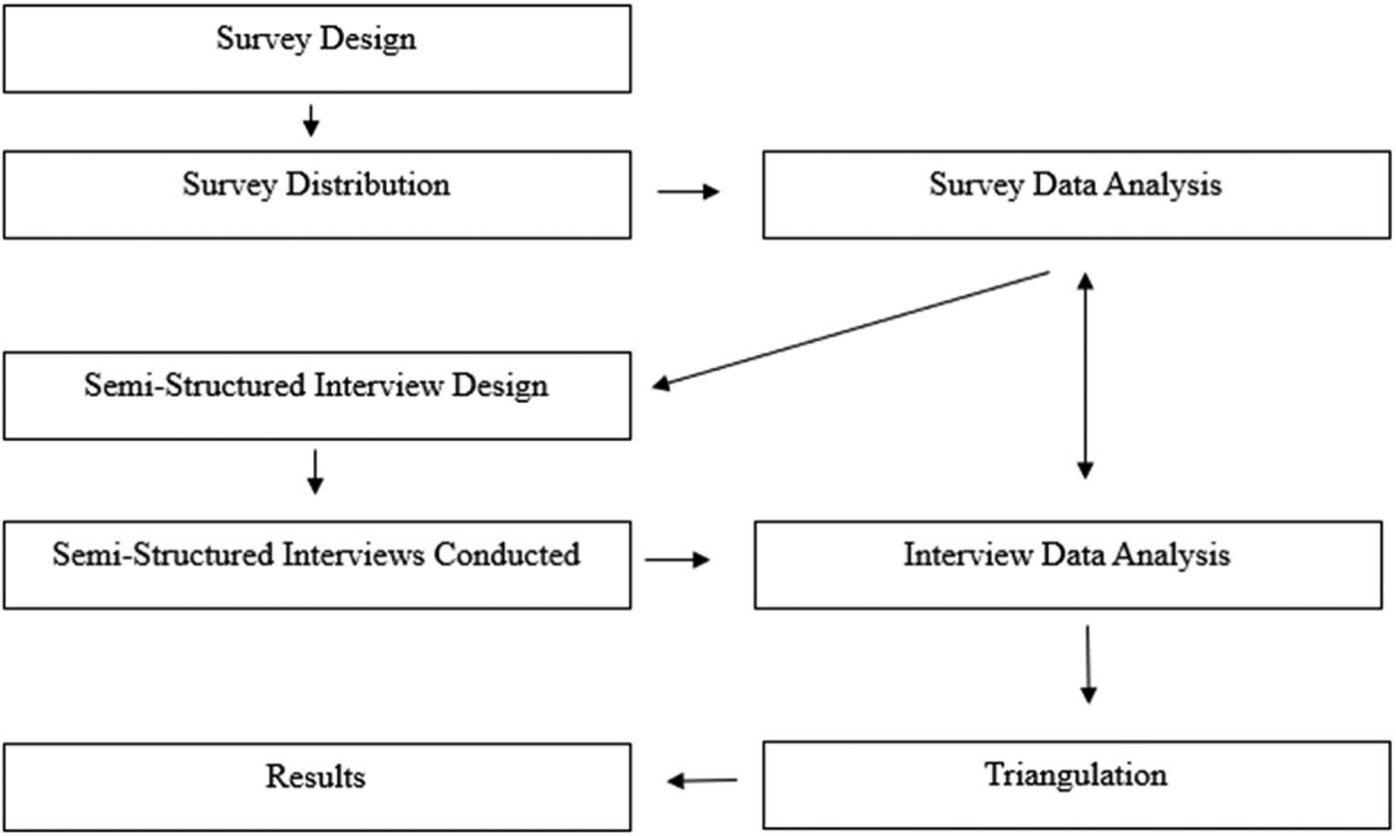
Sequential explanatory design.

A mixed methods approach was selected for this study due to its ability to comprehensively explore this multi‐faceted and highly complex topic—professional training and AAC (Creswell 2014; Creswell and Plano Clark 2018). The explanatory sequential procedure described by Creswell and Plano Clark (2018) was applied and has been outlined in Figure 1. A sequential explanatory mixed methods design consists of two or more phases that occur in a set chronological order, with each phase relying on the previous phase (Tashakkori et al. 2021). This design commences with a quantitative phase (i.e., survey) and investigates specific results with a qualitative phase (i.e., semi‐structured interviews). Following this design, the data obtained from the interviews can be used to add depth, richness, and further explanation of the survey findings (Creswell and Plano Clark 2018; Tashakkori et al. 2021). Therefore, this approach can also be used to explain novel or unusual findings within the quantitative phase of the study (Creswell 2014).

A sequential explanatory design was chosen because (a) it allowed for a clearly identifiable quantitative and qualitative phase to capture the strengths of both methods (Creswell 2014); (b) the main variables present within the topic were known (through the clinical experience of the researcher and through similar surveys completed internationally); and (c) the authors had the ability to access participants for two rounds of data collection (Creswell and Plano Clark 2018).

### 2.2 Positionality

Due to the qualitative component of this mixed methods study, the positionality of each author was considered pertinent due to the subjectivity of the analysis process (Saldana 2021). At the time of this study, Author 1 was a qualified and registered SLP undertaking their PhD. They completed their SLP training in Australia. In addition to teaching AAC to undergraduate SLPs as part of a university programme in Australia, Author 1 continued to provide clinical services to children with disabilities, including AAC users. Author 2 had extensive experience in qualitative research in public health with no prior exposure to the field of AAC. Author 3 was a qualified and registered SLP in Australia who completed their SLP training in Canada. Author 3 had extensive experience conducting quantitative and mixed methods research.

### 2.3 Participants

Participants in both the survey and semi‐structured interviews were SLPs who completed their qualifications through an Australian university and currently worked with paediatric or mixed (paediatric and adult) client caseloads. Participants needed to be eligible to be registered with Speech Pathology Australia and currently employed as a SLP in Australia. SLPs trained at international universities were excluded from this study due to the focus on university training experiences for SLPs in Australia. ‘Paediatric population’ was defined as working with clients aged 0 to 18 years for any proportion of the SLP's caseload. All paediatric SLPs were selected for participation (rather than just those working in AAC) to capture the voices of those SLPs who may not be working with AAC users due to a lack of training in AAC.

Of the 234 survey responses received, 29 were excluded due to not meeting participant eligibility requirements. For respondents who met inclusion criteria, partial survey responses were retained, resulting in the analysis of 205 surveys. These participants were estimated to represent approximately 2.8% of paediatric SLPs in Australia. In 2023, Speech Pathology Australia reported there were 12 185 certified practising SLPs. While the Speech Pathology Australia workforce report outlined that more than half of SLPs work with children, no specific data was provided. For the purpose of this research, ‘more than half’ was estimated at approximately 60% (*n* = 7311).

Demographic information for survey respondents has been provided in Table 1. Following the survey, 16 participants completed individual semi‐structured interviews. Participant profiles have been outlined in Table 2.

**TABLE 1:** Demographic information (survey).

Item	Sub‐item	# (%)
Geographical location	Rural/Remote	90 (44%)
	Metropolitan	114 (55.6%)
	Did not respond	1 (0.5%)
Workplace^a^	Private practice	98
	Non‐government/Not for profit	78
	Hospital or health service	94
	Education department	19
	Other	3
Years’ experience	1–5 years	98 (47.9%)
	6–10 years	54 (26.3%)
	11–15 years	23 (11.2%)
	> 15 years	30 (14.6%)
AAC users on paediatric caseload	0%	8 (3.9%)
	< 20%	42 (20.5%)
	21%–40%	72 (35.1%)
	41%–60%	54 (26.3%)
	61%–80%	19 (9.3%)
	81%–100%	10 (4.9%)

^a^ Participants were asked to select all workplaces where they were currently employed.

**TABLE 2:** Participant profile (semi‐structured interviews).

Participant #	Caseload	Years of experience	% AAC users on caseload	Geographical location
1	Paediatric	2–5 years	21%–40%	Metropolitan
2	Paediatric	2–5 years	61%–80%	Metropolitan
3	Mixed	6–10 years	21%–40%	Metropolitan
4	Paediatric	6–10 years	21%–40%	Metropolitan
5	Paediatric	2–5 years	41%–60%	Metropolitan
6	Mixed	11–15 years	41%–60%	Metropolitan
7	Mixed	6–10 years	21%–40%	Metropolitan
8	Paediatric	6–10 years	41%–60%	Rural
9	Mixed	2–5 years	< 20%	Rural
10	Paediatric	6–10 years	0%	Metropolitan
11	Paediatric	> 15 years	41%–60%	Rural
12	Mixed	6–10 years	81%–100%	Rural
13	Mixed	6–10 years	81%–100%	Metropolitan
14	Paediatric	> 15 years	< 20%	Metropolitan
15	Mixed	11–15 years	< 20%	Rural
16	Paediatric	> 15 years	< 20%	Metropolitan

### 2.4 Survey Design and Distribution

The first author developed a survey to investigate the knowledge, attitudes, confidence, competence, and training experiences of SLPs in AAC as part of a larger study (Conlon and Zupan 2024; Conlon et al. unpublished manuscript); the survey was then reviewed by the second and third authors. Questions were developed based on previous studies (Armstrong et al. 2000; Chua and Gorgon 2018; Dada et al. 2017; Pampoulou et al. 2018; Sutherland et al. 2005) in addition to the first author's clinical experience in AAC. The survey was piloted by two SLPs and one SLP student to provide feedback on readability, length and any errors present; no changes were required. The results reported in the current study are based on a subset of data from this survey. The remainder of the data covered a range of topics and is therefore reported in separate publications (Conlon and Zupan 2024; Conlon et al. unpublished manuscript) to ensure the concept of training could be reported on comprehensively. The survey questions relevant to this study consisted of 11 demographic questions, 8 Likert scale statements pertaining to pre‐professional training (5‐point scale), 13 Likert scale statements pertaining to desire for further training (3‐point scale), 6 Likert scale statements pertaining to preferred training format (3‐point scale) and one multiple‐choice question pertaining to post‐professional training (see Supporting Information for survey questions). The online survey was created using Qualtrics software and disseminated via Facebook and Twitter, the Speech Pathology Australia digital newsletter, and emails to professional networks. The survey was open from 25 September 2022 to 10 November 2022.

### 2.5 Survey Data Analysis

All survey data were exported into the Statistical Package for Social Sciences (SPSS). All categorical data (e.g., demographic questions) and responses to individual Likert statements (e.g., desire for further information or training in AAC) were analysed using descriptive statistics. Inferential statistics (i.e., one‐way ANOVA) were used to investigate the relationship between adequacy of pre‐professional training and years spent in the workforce.

### 2.6 Semi‐Structured Interview Design and Implementation

At the end of the online survey, respondents were asked if they consented to be contacted for an individual interview. Therefore, the participants of the semi‐structured interviews were a subset of the survey participants (Creswell and Plano Clark 2018). After quantitative analysis of the survey data, it was identified by author 1 that the semi‐structured interviews were needed to further explore (1) university and post‐professional training experiences in AAC, (2) positive versus negative training experiences, (3) current training needs of SLPs, (4) training experiences of SLPs from different geographical locations (i.e., metropolitan or rural/remote) and (5) training experiences of SLPs with different years of experience. These focus areas were reviewed and confirmed by Authors 2 and 3. While the participants available for this phase of the project were limited to those who volunteered, the results of the quantitative survey informed which of these volunteers were then selected for the semi‐structured interview otherwise referred to as ‘theoretical sampling’ (Conlon et al. 2020). These groups included SLPs from different geographical locations (metropolitan and rural/remote) and SLPs with different years of experience. Respondents who provided consent and met these characteristics were then contacted.

An interview protocol was developed by the first author in consultation with the second and third author. The interview protocol consisted of one open ended question pertaining to work experience and four open‐ended questions pertaining to training (see Supporting Information). Further questions were asked pertaining to competency; however, these have been reported as part of the companion paper (Conlon et al. unpublished manuscript).

Semi‐structured interviews (*n* = 16) were conducted online via Zoom. An individual interview format was selected to ensure SLPs felt comfortable in expressing their subjective experiences and point of view (Creswell 2014). An online format was selected to reduce the logistical barriers present when scheduling time to meet with a busy SLP and to ensure participants from across a large geographical area (i.e., Australia) could be included.

### 2.7 Data Analysis of Semi‐Structured Interviews

Abductive analysis was used for research questions 1, 3 and 4 to allow the researcher to develop provisional results (based on the quantitative survey) and then further explore and explain these results through the semi‐structured interviews while still being open to any new or surprising phenomena within the data (Palmer and Coe 2020). Abduction seeks to discover new concepts or ideas through a flexible analytic process whereby analysis of data may support and/or suggest modification to current theoretical ideas, hypotheses, or knowledge (Flick 2019; Palmer and Coe 2020). Research question 2 was introduced after the quantitative survey was completed and followed an inductive thematic analysis approach.

The analysis procedure was based on the steps described by Creswell (2014), with further specifications included to suit an explanatory‐sequential design (Creswell and Plano Clark 2018). Step 1: following each interview, the first author wrote a reflection regarding any interesting themes or ideas that had emerged (Saldana 2011). Step 2: the first author transcribed each of the 16 interviews verbatim and uploaded them to NVivo for analysis. Step 3: the first and second authors inductively coded the same three interviews independently and then met to ensure intercoder agreement or consistency was achieved (Creswell and Plano Clark 2018). As part of this process, the first and second authors discussed any themes they felt were initially apparent and how these results aligned or did not align with the quantitative data. Based on the initial three interviews and quantitative data, Authors 1 and 2 proposed an initial set of codes. Step 4: the first author coded the remaining 13 interviews using abductive analysis. Therefore, the initial codes and themes developed in Step 3 were used as a guide, but further codes and themes were added and refined/re‐organised. Step 5: codes were imported into the ‘visualisation’ feature of NVivo, where they were colour‐coded and visually collapsed into themes. This process ensured no codes were missed and any outlier codes could be easily identified. Step 5 was repeated for each of the following concepts: (1) university training experiences, (2) training needs for SLP students and (3) post‐professional training experiences/needs. Step 6: this process was depicted via a series of mind maps which detailed each code that was generated and how the codes were collapsed into themes (see Supporting Information). These mind maps were reviewed and confirmed by Authors 2 and 3. The titles of each theme were also discussed and reviewed to ensure that they captured the essence of the interview theme and could be understood by a wide audience.

It should be noted that while these steps were followed where possible, the iterative nature of abductive analysis meant the researcher moved fluidly back and forth between data collection and analysis throughout the research process (Tashakkori et al. 2021). For example, based on the structure of the semi‐structured interview guide (see Supporting Information), codes for interview questions 3 (describe a positive training experience you have had in relation to paediatric AAC) and 4 (describe a negative or less helpful training experience you have had in relation to paediatric AAC) were originally organised under ‘negative training experiences’ and ‘positive training experiences’. However, when coding the data, it was identified that these aspects (positive and negative) were not clearly delineated, and SLPs predominately described post‐professional training experiences (rather than both university and post‐professional). Therefore, these codes were collapsed into and analysed with ‘post‐professional training experiences’, and a broad research question was created (what are the post‐professional training experiences of SLPs in AAC?) to capture both the quantitative and qualitative data collected.

### 2.8 Triangulation

In explanatory mixed methods designs, findings from the qualitative data assist in explaining and interpreting findings from the quantitative study through triangulation/convergence (Creswell and Plano Clark 2018). Triangulation followed seven steps: (1) Author 1 reviewed the quantitative data for the target research question; (2) Author 1 reviewed the qualitative data for the target research question; (3) Author 1 identified similarities, differences, links and gaps between the quantitative and qualitative data and made notes in a Microsoft Word document; (4) Author 1 identified the most appropriate way to depict the results (e.g., within text or joint display); (5) Author 1 summarised the results using the method identified in Step 4; (6) all authors reviewed the results and discussed representativeness of the data; (7) Author 1 made any changes decided upon by the research team to ensure the triangulation and reporting of data represented the sample. These steps were followed for research questions 1, 3 and 4.

Triangulation of research question 1 was completed in the form of a joint display (Guetterman et al. 2015) to provide depth to the quantitative data in one visualisation. For example, in research question 1, SLPs rated their pre‐professional training as low; therefore, the qualitative themes and subsequent quotes which explain why these ratings were provided by participants are depicted side by side in the joint display. Research question 2 (what AAC training experiences should SLP students receive at Australian universities?) was introduced after analysis of the survey indicated SLPs perceived their own university training experiences to be poor; therefore, the results for this question were qualitative only. Data for research questions 3 and 4 were triangulated and described within the body of the results section to ensure qualitative data that aligned and did not align with the quantitative results could be described (e.g., additional training avenues) in addition to providing explanations for some of the quantitative results (e.g., why SLPs might prefer face‐to‐face AAC training).

### 2.9 Trustworthiness

Data rigour (in qualitative terms) or data validity (in quantitative terms) aims to establish the trustworthiness of the study conducted; however, quantitative, qualitative and mixed methods research establish trustworthiness using different methods (Thomas and Magilvy 2011). Therefore, Appendix 1 outlines how trustworthiness was ensured for the quantitative components of this study according to O'Leary (2021). Appendix 2 outlines how trustworthiness was ensured for the qualitative components of this study according to Creswell and Plano Clark (2018) and Thomas and Magilvy (2011). Appendix 3 outlines how trustworthiness was ensured for mixed methods as the overarching methodology according to the 10 criteria recommended by Tashakkori et al. (2021). Furthermore, this study was reported against the Good Reporting of a Mixed Methods Study (GRAMMS) (O'Cathain et al. 2008) (see Appendix 4).

## 3 Results

### 3.1 Question 1: What Are the University Training Experiences of Australian SLPs in AAC?

On a scale of 1 (none) to 5 (very good), participants were asked, ‘How would you rate the AAC training you received in the following content areas as part of your pre‐professional education?’. As outlined in the joint display shown in Table 3, the mean score indicated that training received in all AAC topics was below adequate except evidence‐based practice (*M* = 3.00, SD = 1.13). Assessment procedures (*M* = 2.68, SD = 1.17) and feature matching (*M* = 2.68, SD = 1.17) received the lowest ratings. However, a standard deviation of 1.13 to 1.27 across all areas indicated high variability in responses, with individual scores ranging from 1 to 5.

**TABLE 3:** Pre‐professional (university) training in AAC.

Survey results				Interview themes
Item	*n*	Mean (SD)	Range	
Assessment procedures	205	2.68 (1.17)	1–5	Insufficient training was received at university to prepare for the workforce ‘*I don't think anyone comes out of university really well trained in AAC*…’ (P14)
Prescription procedures	204	2.74 (1.20)	1–5	‘…*I think it was probably one week that we maybe covered it, and it didn't really*
Feature matching	205	2.68 (1.17)	1–5	*go into a massive amount of depth*’ (P12)
Vocabulary Selection and Organisation	204	2.79 (1.21)	1–5	AAC training experiences do not align with the needs of the contemporary workforce
Training clients to use system	205	2.73 (1.27)	1–5	‘*I feel like it was all centred around supporting people like after they had had a stroke or like with the trachea…it wasn't sort of about supporting kids at all*’ (P1)
Evidence‐based practice	205	3.00 (1.13)	1–5	More practical experiences are needed in university programs
Measuring progress	205	2.82 (1.22)	1–5	‘*I didn't have any placements that had AAC*’ (P2)
Training communication partners	205	2.80 (1.23)	1–5	‘… *there are barriers then in people having placements with people that use AAC so that they might not even know to seek a job or to be interested in that area*’ (P13)

*Note*: Survey participants were asked to respond to the question: How would you rate the AAC training you received in the following content areas as part of your pre‐professional (i.e., university) education? 1 = none, 2 = limited, 3 = adequate, 4 = good, 5 = very good.

The findings of the semi‐structured interviews aligned with the results of the survey, with three themes providing further information regarding why most SLPs felt their pre‐professional training was inadequate: (1) Insufficient training was received at university to prepare for the workforce; (2) AAC training experiences do not align with the needs of the contemporary workforce; and (3) More practical experiences are needed in university programmes. Generally, SLPs felt that they had been given minimal training in AAC, *‘so that was just the one tutorial… it was informative, but it was just not enough’* (P9), which resulted in the need for substantial post‐professional training. Some SLPs felt that universities were not prioritising enough time for AAC within the curriculum: *‘I sometimes wonder whether universities are focused too much on the easy communication disorders like your speech sound disorder kids… they can be tricky, but I don't think that enough time is put into those really complex disorders…’* (P14). For those participants who had received training in AAC, many felt that the unit did not align with workforce needs. Finally, participants emphasised the need for clinical placements to bridge the gap between theory and practical experiences; however, they also acknowledged that placement experiences vary based on the site and the clinical educator: *‘it's whoever your, you know, the clinical educator at the time… may or may not be experienced with AAC, so you're not getting the exposure’* (P16). Those who had placements in AAC, described the positive impact it had on their learning: *‘When I did my final placement, I got a scholarship to do it with the Cerebral Palsy Alliance, so I got to see AAC when I was on placement there… and my supervisor was a bit of an AAC guru, so I got to see lots of different systems for lots of different people, which I think was really helpful for me in not feeling so scared…’* (P1).

Likert scale statements for each pre‐professional training topic (see Supporting Information—Question 10) were collapsed into a single domain (adequacy of pre‐professional training) to allow for further statistical analysis. The internal consistency of this domain was tested using Cronbach's alpha reliability coefficient (α = 0.960), indicating good internal consistency and reliability (Morgan et al. 2020). Results of the one‐way ANOVA indicated ratings of adequacy of pre‐professional training did not significantly differ by years spent in the workforce, *F*(4, 198) = 1.83, *p* = 0.124. This was commensurate with findings from the semi‐structured interviews whereby reporting of positive versus negative training experiences at university did not appear to differ based on years spent in the workforce. Only two participants reported receiving comprehensive training in AAC as a stand‐alone unit. Although they differed in years of experience (> 15 years versus 6–10 years), they both reported having their unit taught by the same Australian academic who has extensive experience in AAC.

### 3.2 Question 2: What AAC Training Experiences Should SLP Students Receive at Australian Universities?

When describing the AAC training experiences SLP students should receive at Australian universities, three main themes were identified by the authors: (1) theoretical content specific to AAC needs to be explicitly taught; (2) students require practical experiences to understand AAC; and (3) AAC should be comprehensively taught either as a stand‐alone unit or embedded across other units.

#### 3.2.1 Theoretical Content Specific to AAC Needs to be Explicitly Taught

SLPs felt that students needed to know the theory that underpins AAC, especially in topics that are unique to AAC. For instance, SLPs described the nuances of assessment for AAC users, which SLP students tend to struggle with due to the need for informal assessments, *‘I do tend to find, like students come out of uni [university] and it's just about formal assessment, formal assessment, formal assessment and I would love to see them be able to do some of that informal stuff’* (P12). As part of the assessment process, SLPs felt students also need to understand how to feature match against a range of AAC systems. Participant 8 suggested *‘possibly a unit on how to kind of feature match, how to specifically research these devices and how that can be incorporated where AAC is the focus’*. Knowledge of the feature matching process is a key component of conducting AAC trials, which Participant 6 identified as an important process for SLP students to understand, *‘…where they have to prescribe a system and set therapy goals…specifically for a trial. I think that's really important given how it affects clients funding, it affects their ability to access appropriate systems… you just need to know how to set goals and measure the progress appropriately so that you can decide if this trial has been successful or not’*. SLPs also highlighted the importance of students knowing where to find more information given the fast changing landscape of AAC, *‘I guess the main thing is technology and communication systems change a lot, so what students might learn right now, today, in five years’ time is probably going to be quite different, so learning the nitty gritty [the details]…I don't think that's as relevant as learning how to seek information’* (P13).

#### 3.2.2 Students Require Practical Experiences to Understand AAC

The impact of practical experiences for SLPs and SLP students was apparent throughout the interviews. SLPs highlighted that students require practical experiences within the academic context (e.g., guest lectures from AAC suppliers and hands‐on training with devices), in addition to placement experiences with AAC users. Participant 9 acknowledged there were many barriers to universities accessing AAC resources but *‘… you know if it was a perfect world, having those resources constantly available within the university spaces, so that clinicians or students can go in and practice with all of them’*. Participant 1 highlighted that hands‐on experience is vital to *‘… get some actual, real experience beyond hearing words about it. What does it look like? How do we actually do it?’* The concept of practical experiences connected with some SLPs’ views that students also needed placements in a disability context. Participant 12 went as far as to say that *‘… the ideal would be to have a dedicated placement for every university student where they would have to do something with AAC’* but recognised this may not be realistic due to the restraints of placement offers.

#### 3.2.3 AAC Should be Comprehensively Embedded Across Other Units or Taught as a Stand‐Alone Unit

Some participants believed that AAC should be taught as a stand‐alone unit, *‘I think if you could have an AAC subject… that would be amazing to get all that experience and really focus on that practical goal setting, implementing in a real hands on way’* (P14). Other participants believed that AAC should be embedded across a speech pathology course to ensure students saw AAC as an integral part of language intervention, *‘…AAC is also language, it's not separate from language, you know’* (P13). In contrast other participants felt that ideally AAC should be comprehensively taught within a stand‐alone unit in addition to being embedded across other units. Participant 16 really captured why this was perceived to be important, *‘I think AAC needs to be infused into every part of the curriculum…maybe a separate subject that is focused on it would also be great… I think that* [not having a subject on AAC] *can give students the impression that it's something I can either specialise in and do, or I cannot do… the stuttering subjects, you know you take it or leave it, AAC isn't a take it or leave it’*.

### 3.3 Question 3: What Are the Post‐Professional Training Experiences of Australian SLPs in AAC?

In the quantitative survey, SLPs were asked to indicate where they have received knowledge, skills, or training in AAC since completing their university training. Journal articles were identified most frequently (*n* = 156), followed by websites (*n* = 149), books (*n* = 135), in‐person workshops (*n* = 133), social media (*n* = 124) and live online workshops (*n* = 107). Mentoring (*n* = 90), pre‐recorded workshops/webinars (*n* = 85), external mentoring (*n* = 77), certificate courses (*n* = 71) and conferences (*n* = 61) were identified least frequently.

When asked to describe their training experiences in AAC, interview participants reported accessing training through six main sources: on‐the‐job experience, AAC companies, online training, mentoring/supervision, conferences and overseas training. SLPs often described learning through on‐the‐job experience due to a lack of training offered by their employer. For example, Participant 8 shared, *‘I started working at [name of company and town] and yeah, they didn't have a lot of supervision, so it was just a lot of like trial and error’*. Most SLPs positively described training received from companies who specialise in AAC, such as AAC suppliers (e.g., Liberator, Link Assistive and Zyteq) and the Australian Group Supporting Communication Inclusion (AGOSCI). These SLPs described a range of training received through these companies, including formal (face‐to‐face) training, online training, and advice/mentoring. However, some SLPs felt that training offered by AAC suppliers could generate bias towards certain systems, ‘*I think that's been the biggest, I guess peeve* [annoyance] *that I've had with all of my training, has been that bias with, you know, because the companies doing the training, are selling the products a lot of the time’* (P7). SLPs also felt this bias existed if they had a supervisor who had a preference for a certain AAC system, such as Participant 1, *‘She* [the senior] *had just learned like we do this one system and that was it, so then, if I sort of asked questions or was like we should we try, and you know, do this instead… then she was quite resistant so I found that hard to learn from…’*.

### 3.4 Question 4: What Are the Training Needs of Australian SLPs in AAC?

**FIGURE 2:**
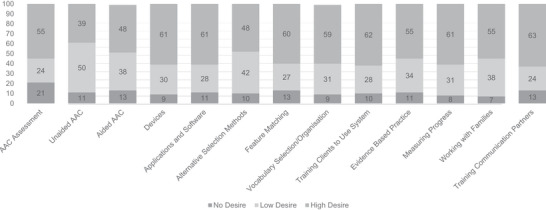
Desire for further information or training by topic *Note*: Each value represents the percentage of SLPs who provided the associated response.

Overall, participants indicated a desire for further training in all topics presented in the survey question. This is shown in Figure 2, which identifies the percentage of SLPs who selected 1 (no desire), 2 (low desire) or 3 (high desire) for each topic. Training for supporting clients to use their AAC system (*M* = 2.52), training communication partners (*M* = 2.51) and different AAC options/devices (*M* = 2.51) received the highest ratings; unaided symbol sets/systems received the lowest rating (*M* = 2.28).

When asked about their preferences for training formats, SLPs indicated that they ‘preferred’ or ‘strongly preferred’ mentoring from an AAC specialist (*n* = 180), followed by in‐person workshops (*n* = 177), self‐paced learning modules (*n* = 169), recorded webinars (*n* = 167) and live webinars (*n* = 160); reading materials received the lowest ratings (*n* = 147).

A preference for in‐person workshops and contact with an AAC specialist were strong themes within the interviews, as evidenced in responses to the questions that asked them to describe a positive and negative/less helpful training experience. Interview participants reported a preference for training that is practical, face‐to‐face, and presented by someone who is knowledgeable and passionate about AAC. Participants felt training in this format was more engaging, which made it easier to focus. It also gave them the opportunity to talk to their peers and increased confidence in their own skills and knowledge. Conversely, SLPs had mixed experiences with online training and felt it lacked practical application. In addition, they shared it was often not specific to SLPs and/or the content was too basic.

## 4 Discussion

This study found that Australian SLPs felt their university training in AAC did not prepare them for the workforce, and this did not differ by years spent in the workforce. Participants reported that universities should be providing AAC training to SLPs that is explicit, practical and comprehensive. While participants in this study reported accessing a range of post‐professional training avenues, they indicated a high desire for further AAC training across all surveyed topics. SLPs indicated they preferred AAC training that was practical, face‐to‐face and presented by someone knowledgeable in AAC, such as in‐person workshops and mentoring from an AAC specialist. These results within the context of the broader literature have been discussed forthwith.

### 4.1 University Training Experiences and Needs of SLPs in AAC

The Augmentative and Alternative Communication Guideline published by Speech Pathology Australia in 2012 stated that ‘the inclusion of multimodal communication as a Range of Practice in the CBOS has resulted in the need to incorporate relevant information in professional training’ (Speech Pathology Australia 2012, 28). This documentation highlights that the introduction of MMC as a practice area in 2011 should have increased the presence of AAC content within Australian SLP university programmes. However, the results of the current study suggest that either (1) AAC is still not comprehensively taught within university programmes in Australia or (2) AAC is being taught, but the content is not aligned to the needs of the contemporary workforce. While this is a complex and multifaceted topic, two potential reasons for a lack of AAC training at Australian universities have been proposed: (1) person‐driven versus policy‐driven curriculum design and (2) needs of the contemporary workforce.

Curriculum design within universities is complex and driven by many interrelated factors, including internal university policies, external policies, accreditation bodies (such as Speech Pathology Australia) and the staff employed to develop the curriculum. Australia is fortunate to have policies and external accreditation requirements to influence university programmes to teach AAC. For example, Speech Pathology Australia's Augmentative and Alternative Communication Guideline states that ‘Academics in speech pathology courses in Australia are working to incorporate all elements of competence in multimodal communication, including both theoretical and clinical experience required for practice’ (Speech Pathology Australia 2020a, 28). However, the results of this study show this recommendation has not eventuated (yet), even for recent graduates, suggesting that there are other factors influencing curriculum decision‐making, such as staff profiles. In the data presented here, the outliers were two SLPs interviewed who reported receiving comprehensive training in AAC; this data did not align with responses of the other SLPs. Interestingly, although their time spent in the workforce differed, both SLPs reported being taught by the same academic who specialises in AAC. This suggests that decision‐making around curriculum content could be heavily person‐driven rather than just policy‐driven. Between 2008 and 2018, the United States reported an increase in speech pathology programmes offering at least one course in AAC (2008 = 73%; 2018 = 86%), which happened alongside a significant increase in academics with primary expertise in AAC (2008 = 29%; 2018 = 81%) (Johnson and Prebor 2019). Unfortunately, determining teaching personnel in university programmes for specific areas is difficult through publicly accessible documents, so an exploration into this premise was not possible as part of the current study. However, it seems plausible that a lack of academics in the field of AAC may be a contributing factor to the state of AAC training in Australia. Specific investigation into this factor is needed to further understand the influence of person‐driven factors on the curriculum design of SLP programmes.

Some SLPs felt that university programmes focused too heavily on speech and language cases for verbal children rather than on complex presentations such as clients who use AAC. This could represent a disconnect between university education and the needs of the modern workforce. Traditionally, clients with disabilities were predominately seen by government organisations that specialised in disability, such as Disability Services Queensland. However, the introduction of the NDIS in 2013 has meant that more clients with disabilities now have access to funding and that funding is used within private and non‐government services. In fact, private practices are now the most common employer of SLPs in Australia (Speech Pathology Australia 2023). This represents a shift in what could be viewed as a ‘typical’ caseload for an Australian SLP. Respondents in the current study support this premise; it was targeted at all paediatric SLPs (regardless of caseload), and 96.1% of participants reported they were currently supporting AAC users. This means that all SLP students in Australia need to be prepared to support AAC users upon entering the workforce; as aptly put by an interview participant, *‘AAC isn't a take it or leave it’*.

### 4.2 Post‐Professional Training Experiences and Needs of SLPs in AAC

The top three sources of post‐professional training or information about AAC represented a mismatch between the training SLPs are acquiring and their training preferences: practical training, preferably face‐to‐face, delivered by someone passionate and knowledgeable about AAC. The desire for practical AAC training is consistent with international literature (Gohsman and Johnson 2023). Journal articles, websites and books are most likely more common avenues for training and information sharing given their low cost and an SLP's ability to access these resources in their own time. While these options are convenient, incompatibility between a health professional's individual learning needs and the format of training offered may impact on the effectiveness of that training in relation to practice‐based outcomes (Berndt et al. 2017). More importantly, it may impact the SLP's confidence to implement this learning in their clinical practice given that clinical confidence is linked to clinical skills specifically in the area of AAC (Conlon et al. 2024).

SLPs indicated a strong preference for face‐to‐face learning, but comparatively, preferences for different types of online learning were mixed. While at surface level, online training options have comparable outcomes to their face‐to‐face counterparts for allied health professionals (Berndt et al. 2017), this may not be generalisable to AAC due to the hands‐on nature of the field. When discussing both university training and post‐professional training, SLPs in this study emphasised the need for hands‐on learning with AAC systems, which is difficult to facilitate during most online learning formats. While a simple recommendation would be for AAC training to be offered in person only, this creates significant logistical barriers for clinicians living in rural and remote areas (Curran et al. 2006); rural and remote participants comprised 44% of this survey sample. Another consideration would be to offer training in a mixed‐mode format, whereby theory is taught online and practical content is taught face‐to‐face. This would reduce costs associated with time and travel for rural and remote SLPs. Mixed mode is becoming a popular training format for undergraduate allied health courses (Naing et al. 2023); however, application to post‐professional training has not yet been thoroughly investigated. Therefore, further research into the specific training preferences of SLPs is warranted. This should include an update on the work completed by Beukelman et al. (2005), which takes into consideration current technological advances in training.

This study also highlighted the need for SLPs to access professional supervision from someone with extensive experience and knowledge in AAC. This need aligns with recommendations from Speech Pathology Australia for SLPs to access professional support, such as mentoring or supervision, throughout their careers (Speech Pathology Australia 2022). However, the limited use of mentoring reported by participants in this study was often ascribed to workplace restrictions (e.g., supervision was not offered) and/or difficulty sourcing an SLP with experience in AAC for supervision. This was where AAC suppliers were highlighted as a vital resource in Australia, as they hire SLPs who work solely in the area of AAC. Some companies, such as Liberator, were reported to offer free mentoring to SLPs working through the feature matching process, which circumvents the issue of convincing employers to finance external supervision, which SLPs identified as a barrier to developing competence (Conlon et al. unpublished manuscript).

## 5 Limitations and Future Research Directions

In order to obtain a range of perspectives, this survey was open to all paediatric SLPs who trained in Australia. Despite this, only 3.9% of survey respondents did not work with AAC users. It is important to note, though, that for most respondents (55.6%), AAC users comprised the minority of their caseload (< 40%). Since the advertisement for the survey mentioned AAC, SLPs who worked with AAC users (even minimally) may have been more likely to complete the survey. Alternatively, given the impact of the NDIS increasing funding for clients with disabilities, this could be representative of the proportion of SLPs now servicing AAC users. This study was also limited to SLPs with a paediatric or mixed caseload and therefore excluded SLPs working exclusively with the adult population. It is recognised the SLPs working with adults could add valuable insight into training experiences and needs in AAC. Therefore, caution should be taken when generalising these results to the population of SLPs as a whole across Australia.

The interview guide did not include questions to explore the barriers and facilitators to accessing training for SLPs, which would have been useful in understanding how key stakeholders can support SLPs to access further post‐professional training in AAC. Furthermore, this research has highlighted that there are possible discrepancies in how AAC is being taught in university programmes across Australia, suggesting a possible disconnect between the content taught and the contemporary workforce. Therefore, future research should seek to explore what training is currently occurring in Australian university programmes similar to ongoing research conducted in the United States (Johnson and Prebor 2019; Sauerwein and Burris 2022).

## 6 Conclusion

Nearly all paediatric SLPs who took part in this survey provided support to AAC users. However, these SLPs felt the training they had received at university was inadequate, and there are many clinical skills that are unique to AAC which cannot be directly applied from other ranges of practice areas. Therefore, SLPs recommended that certain AAC topics should be explicitly taught at university utilising theory and practical learning. This includes assessing individuals who cannot use speech alone to be heard and understood, feature matching and completing AAC trials and prescriptions. Given these factors, it is imperative that university curricula incorporates more comprehensive training in AAC. Although SLPs will always need post‐professional training, early career SLPs entering the workforce need to have strong foundational skills in AAC, so that they possess both the necessary competence and confidence to start working in this area.

## Ethics Statement

This study was approved by the Central Queensland University Human Research Ethics Committee (reference number 23676).

## Consent

Participant consent was received from all research participants as per the procedure approved by the Central Queensland University Human Research Ethics Committee (reference number 23676).

## Conflicts of Interest

The authors declare no conflicts of interest.

## Permission to Reproduce Material from Other Sources

No permission is required to reproduce materials from other sources.

## Supporting information




**Supplementary information**: jlcd70111‐sup‐0001‐SuppMat.docx

## Data Availability

The data that support the findings of this study are available on request from the corresponding author, [CC]. The data are not publicly available at this point in time due to finalisation of the dataset for the first authors PhD thesis. This is to ensure privacy of the research participants, and that the data is made available in accordance with the research data management plan approved by the CQUniversity Human Research Ethics Committee.

## Appendix 1

### Trustworthiness (Quantitative)

**Table:**

Component	Application to study
Internal validity	Surveys from similar studies conducted internationally were used to inform the quantitative survey questions. The survey was piloted to ensure the questions were valid and accurately interpreted by participants. During survey analysis, all assumptions of statistical tests were checked and adhered to. Quantitative data analysis (SPSS files and output) was checked by another author to ensure accuracy.
External validity	The survey was disseminated nationally to maximise representativeness of the sample. A description of pertinent participant characteristics were provided.
Reliability	The purpose of the study was clearly described. The sampling method was clearly described. Cronbach's Alpha Coefficient was used to measure the internal reliability and consistency of survey items.
Objectivity	Purposive (random) sampling was used to recruit participants (anonymously) to the survey to minimise sampling bias.

## Appendix 2

### Trustworthiness (Qualitative)

**Table:**

Component	Application to study
Credibility	Three interviews were transcribed and then coded independently by two authors. Mind maps were reviewed and confirmed by the research team. Each interview transcript was reviewed to identify similarities between participant experiences to identify themes.
Transferability	A description of pertinent participant characteristics were provided. Data saturation was used in addition to a target sample size to fit within the project parameters. Data saturation was met when (1) no new themes were generated and (2) sufficient data had been collected to answer the research questions.
Dependability	The purpose of the study was clearly described. The nested sampling method was clearly described. The data analysis procedure was clearly described. Copies of the mind maps which showed codes and themes were provided as supplementary materials. Codes for three interviews were reviewed and discussed by two authors until agreement was reached. This process was used to ensure reliability of coding while also acknowledging the importance of individual researcher perspectives. Data outliers were clearly reported in the mind maps.
Confirmability	Throughout the interviews, the first author asked probing questions when limited information was provided or clarity was required to ensure assumptions were not made. After each interview, the researcher wrote a reflection regarding any insights including personal feelings or bias.

## Appendix 3

### Trustworthiness (Mixed Methods)

**Table:**

Component	Application to study
Design suitability	Each research method was specifically chosen based on the research question(s).
Design implementation fidelity	Data collection and sample sizes were carried out based on the method selected. For example, the quantitative component used random sampling and a G‐Power analysis whereas the qualitative component used purposive sampling and sought data saturation.
Within‐design consistency	Surveys and semi‐structured interviews were deemed as appropriate methods to collect data from SLPs as they have the language, literacy and cognitive capability to respond to the target questions independently.
Analytic adequacy	The data analysis techniques were chosen based on each research question and subsequent method.
Interpretive consistency	Consistency was established by ensuring (1) inferences were made based on the type of data (e.g., correlation data was not used to firmly establish cause) and (2) conclusions regarding strength were reported accurately (e.g., if themes only related to the minority of participants, this was stated).
Theoretical consistency	All results were compared to current theories and research data in the discussion section. Situations where the results did not align with theories or current research were clearly stated.
Interpretive agreement	All data analysis, results and the final manuscript were reviewed by all authors to ensure all points of view were considered.
Interpretive distinctiveness	Within the quantitative component, known variables were considered such as geographical location and years of experience. Within the qualitative component, reflexivity was considered, reflected upon and discussed throughout the study between all authors.
Integrative efficacy	The steps taken for data triangulation including who was involved in each step was described. *How* this was achieved was described for individual research questions (e.g., use of a joint display).
Interpretive correspondence	The purpose of using mixed methods research was to gain a comprehensive insight into training in AAC in Australia which required exploration into a range of sub‐topics. The design used has met this need.

## Appendix 4

### Good Reporting of a Mixed Methods Study (GRAMMS)

**Table:**

Guideline	Section (Page)
Describe the justification for using a mixed methods approach to the research question	Method (8)
Describe the design in terms of the purpose, priority and sequence of methods	Method (8, Figure 1)
Describe each method in terms of sampling, data collection and analysis	Method—Participants (8–9) Method—Survey design and distribution (9–10) Method—Survey data analysis (10) Method—semi structured interview design and implementation (10–11) Method—Data analysis of semi structured interviews (11–12)
Describe where integration has occurred, how it has occurred and who has participated in it	Method—Triangulation (12–13).
Describe the limitation of one method associated with the present of the other method	Limitations and Future Research Directions (25)
Describe any insights gained from mixing or integrating methods	Discussion (20–25)
