# Supplementary material for: ‘AAC Isn't a Take It or Leave It’: The Augmentative and Alternative Communication Training Experiences of Australian Speech‐Language Pathologists Working in Paediatrics
Source: Int J Lang Commun Disord. 2025 Aug 23;60(5):e70111. doi: 10.1111/1460-6984.70111 (PMC12374561; doi:10.1111/1460-6984.70111)
Supplement: Supplementary file 1 — Supplementary information: jlcd70111‐sup‐0001‐SuppMat.docx [file JLCD-60-0-s001.docx]

**Supplemental Materials**

**Survey Questions**

1. Please tick the box that best describes where you work and who you work with:

- I currently work as a speech pathologist in Australia and service only paediatric clients (0-18 years)
- I currently work as a speech pathologist in Australia and service a mix of paediatric clients (0-18 years) and adult clients (over 18 years)
- None of the above

1. Please tick the box that best describes you:

- I completed a Bachelor of Speech Pathology through an Australian University
- I completed a Bachelor of Speech Pathology (Honours) through an Australian University
- I completed a Master of Speech Pathology through an Australian University
- I completed my speech pathology qualifications at an International University

1. Are you a member or eligible to be a member of Speech Pathology Australia?

- Yes
- No

1. Where do you currently provide the majority of your services? If you are unsure, you can access the Health Workforce Locator here: https://bit.ly/3yT8ZcY

- Metropolitan
- Rural
- Remote

1. What proportion of your practice is conducted using telehealth?

- 0-25%
- 26-50%
- 51-75%
- 76-100%

1. In which setting/s are you employed? Tick all that apply.

- Private practice
- Non-government/Not for profit organisation
- Hospital or Health Service
- Education Department
- Other

1. How many years have you been working as a speech pathologist?

- <1 year
- 2-5 years
- 6-10 years
- 11-15 years
- >15 years

AAC is defined as communication strategies, techniques, and interventions for people with a range of communication needs involving the use of a system to augment or in addition to verbal output. This may include unaided systems such as gesture or Key Word Sign or aided systems such as communication boards or speech generating devices.

1. Do you currently provide services to children (0-18 years) who use augmentative and alternative communication systems (AAC)?

- Yes
- No

1. What proportion of your caseload (children 0-18 years) are AAC users?

- less than 20%
- 21-40%
- 41-60%
- 61-80%
- 91%-100%

1. The following questions pertain to training you have received at the pre-professional (i.e., University) and post-professional levels (i.e., since entering the workforce).How would you rate the AAC training you received in the following content areas as part of your **pre-professional** (i.e., University) education?

|  | None | Limited | Adequate | Good | Very Good |
| --- | --- | --- | --- | --- | --- |
| Assessment procedures |  |  |  |  |  |
| Prescription procedures |  |  |  |  |  |
| Feature matching (selecting systems that match the client’s needs and skills) |  |  |  |  |  |
| Training clients to use his/her system |  |  |  |  |  |
| Evidence-based practice in AAC |  |  |  |  |  |
| Measuring progress (data collection, measurement of outcomes) |  |  |  |  |  |
| Training communication partners |  |  |  |  |  |

1. Since completing your university training (i.e., **post-professional**), where have you received knowledge, skills, or training in AAC? (tick all that apply)

- Books
- Journal articles
- Websites
- Social media
- Face to face (in person) workshops
- Live online workshops
- Pre-recorded online workshops or webinars
- Conferences
- Certificate courses
- Mentoring from a colleague or supervisor
- Mentoring from an external specialist

1. How much do you desire further training or information on the following topic areas related to paediatric AAC?

|  | No desire | Low desire | High desire |
| --- | --- | --- | --- |
| AAC assessment |  |  |  |
| Unaided symbols sets or systems (e.g., Key Word Sign) |  |  |  |
| Aided symbol sets or systems (e.g., picture- and object-based symbols) |  |  |  |
| Devices (range of options and distributors; device specifications and use) |  |  |  |
| Applications and other software (range of options and programming) |  |  |  |
| Alternative selection methods (e.g. eye gaze, switches) |  |  |  |
| Feature matching (selecting systems the match the client’s needs and skills) |  |  |  |
| Vocabulary selection and organisation |  |  |  |
| Training clients to use his/her system |  |  |  |
| Evidence-based practice in AAC |  |  |  |
| Measuring progress (i.e., data collection, measurement of outcomes) |  |  |  |
| Working with families |  |  |  |
| Training communication partners |  |  |  |

1. If training on this topic were made available to you, what would be your preferred format for that training?

|  | Not preferred | Preferred | Strongly preferred |
| --- | --- | --- | --- |
| Live webinars |  |  |  |
| Recorded webinars |  |  |  |
| Face to face (in-person) workshops |  |  |  |
| Mentoring from an AAC specialist |  |  |  |
| Self-paced learning modules |  |  |  |
| Reading materials (e.g., journal articles, textbooks, information packs) |  |  |  |

**Semi-structured Interview Protocol**

Question 1: Tell me all about your work experience in AAC?

Question 2: Tell me all about your training experiences in AAC?

- Prompt: Tell me all (or more) about your student training experiences in AAC?
- Prompt: Tell me all (or more) about your training experiences in AAC since entering the workforce?

Question 3: Describe a positive training experience you have had in relation to paediatric AAC?

Question 4: Describe a negative or less helpful training experience you have had in relation to paediatric AAC?

Question 5: Reflecting on your experiences as a clinician and your pre-professional training in AAC, describe the training experiences you believe SLP students should receive in AAC.

Prompt: Is there anything else you would like to tell me?

**Mind map – What are the training experiences of SLPs in AAC?**


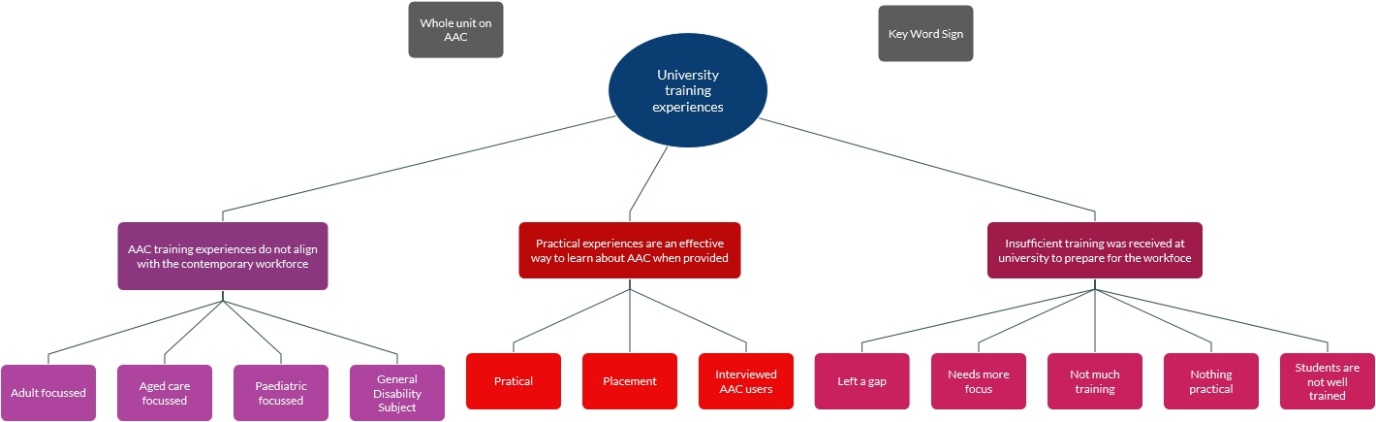


Codes

Themes

Outlier Codes

*Note.* Figure produced in NVivo 12 (QSR International Pty Ltd., 2020)

*Note.* Figure produced in NVivo 12 (QSR International Pty Ltd., 2020)

**Mind map – What AAC training experiences should SLPs receive at university?**


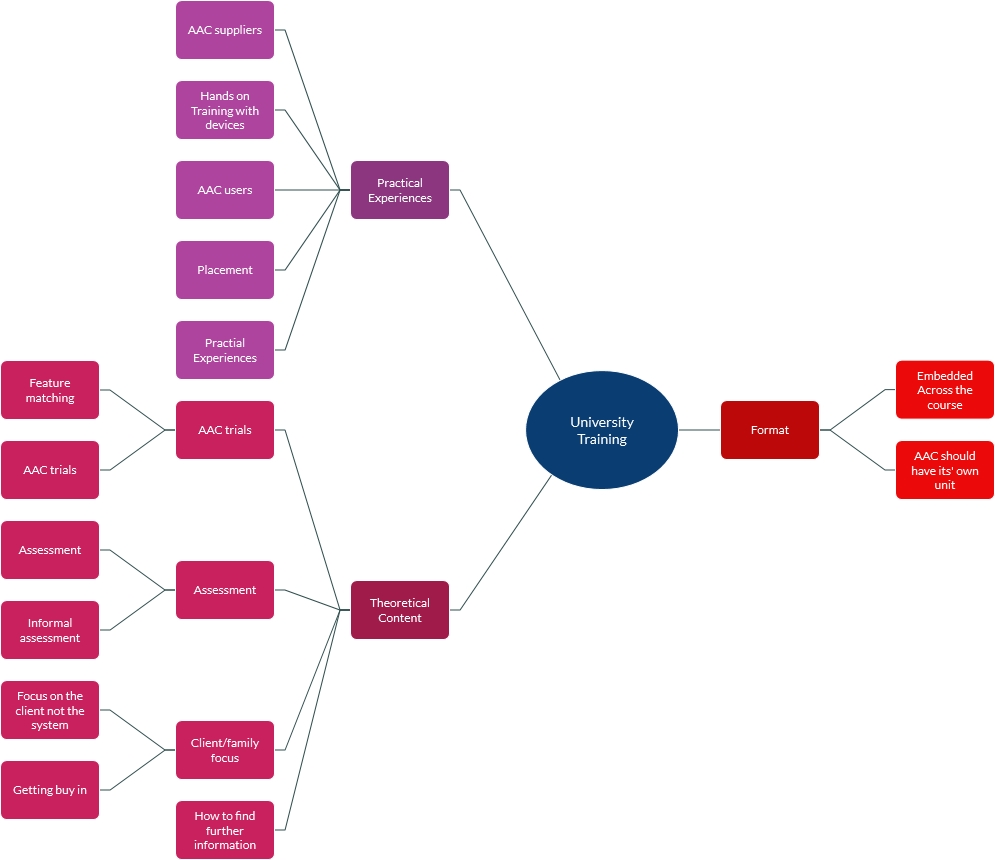


Codes

Themes

*Note.* Figure produced in NVivo 12 (QSR International Pty Ltd., 2020)

**Mind map – What are the post-professional training experiences of SLPs in AAC?**


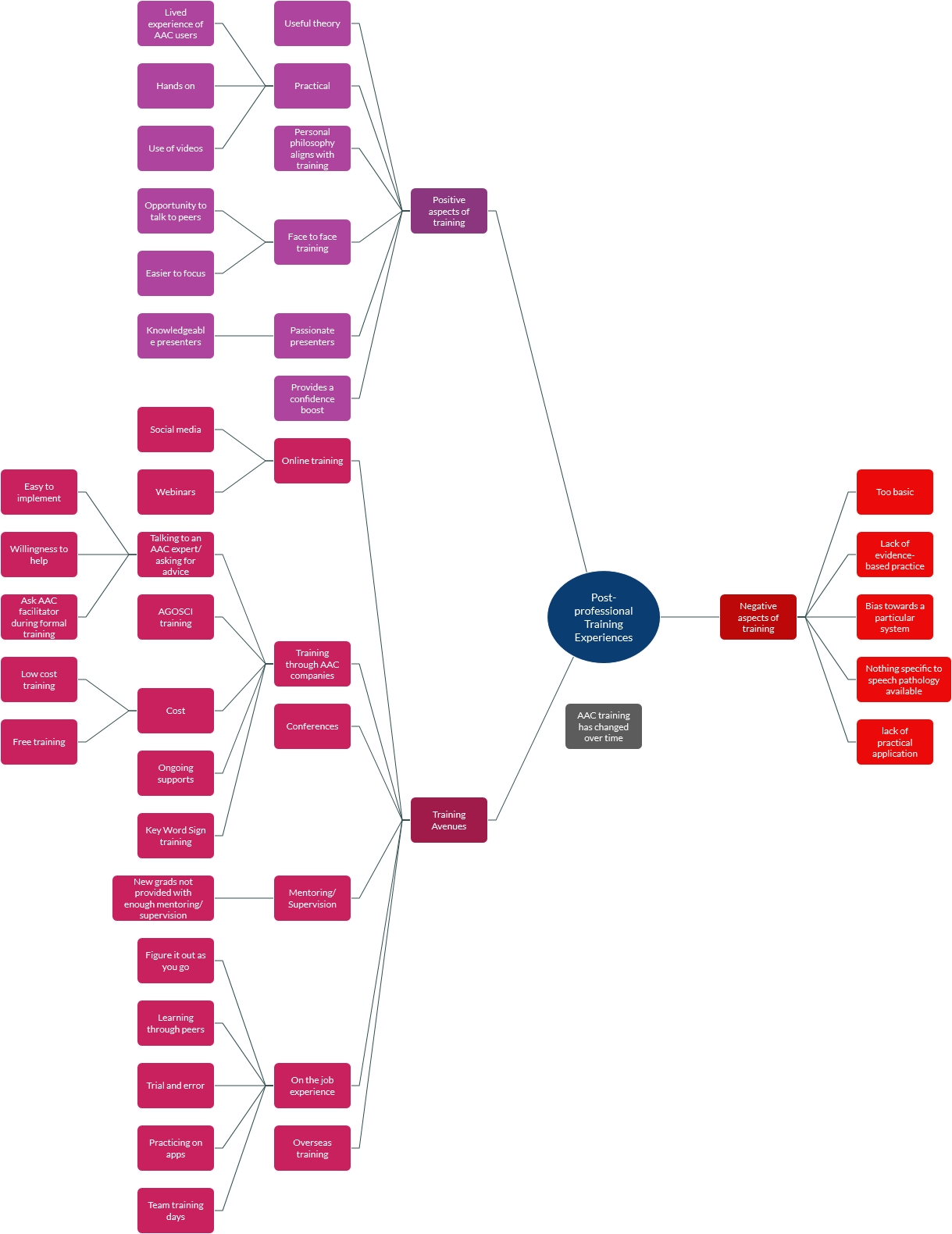


Codes

Themes

Outlier Codes

*Note.* Figure produced in NVivo 12 (QSR International Pty Ltd., 2020)
